# Routine blood tests and probability of cancer in patients referred with non-specific serious symptoms: a cohort study

**DOI:** 10.1186/s12885-017-3845-9

**Published:** 2017-12-04

**Authors:** Esben Næser, Henrik Møller, Ulrich Fredberg, Jan Frystyk, Peter Vedsted

**Affiliations:** 1 Department of Public Health, Research Unit for General Practice, Aarhus University, Bartholins Allé 2, 8000 Aarhus C, Denmark; 2 Department of Public Health, Research Centre for Cancer Diagnosis in Primary Care, Aarhus University, Bartholins Allé 2, 8000 Aarhus C, Denmark; 3 Diagnostic Centre, University Research Clinic for Innovative Patient Pathways, Silkeborg Regional Hospital, Falkevej 1-3, 8600 Silkeborg, Denmark; 4 Cancer Epidemiology and Population Health, Kings College London, Great Maze Pond, London, SE1 9RT UK; 5 Department of Clinical Medicine, Medical Research Laboratory, Aarhus University, Nørrebrogade 44, 8000 Aarhus C, Denmark

**Keywords:** Early cancer diagnosis, Neoplasm, Urgent referral, Primary health care, Denmark

## Abstract

**Background:**

Danish cancer patients have lower survival rates than patients in many other western countries. Half of the patients present with non-alarm symptoms and thus have a long diagnostic pathway. Consequently, an urgent referral pathway for patients with non-specific serious symptoms was implemented throughout Denmark in 2011–2012. As part of the diagnostic workup, a panel of blood tests are performed for all patients referred by their general practitioner (GP) to the urgent referral pathway. In this study, we analysed the probability of being diagnosed with cancer in GP-referred patients with abnormal blood test results.

**Method:**

We performed a cohort study that included all patients aged 18 years or older referred by their GP to Silkeborg Regional Hospital for analysis of a panel of blood tests. All patients were followed for 3 months for a cancer diagnosis in the Danish Cancer Registry. The likelihood ratio and post-test probability of subsequently finding cancer were calculated in relation to abnormal blood test results.

**Results:**

Among the 1499 patients included in the study, 12.2% were subsequently diagnosed with cancer. The probability of cancer increased with the number of abnormal blood tests. Patients with specific combinations of two abnormal blood tests had a 23–62% probability of cancer. Only a few single abnormal blood tests were linked with a high post-test probability of cancer, and most abnormalities were not specific to cancer.

**Conclusions:**

A number of specific abnormal blood tests and combinations of abnormal blood tests markedly increased the probability of cancer being diagnosed. Still, abnormal blood test results should be interpreted cautiously as most are non-specific to cancer. Thus, results from the blood test panel may strengthen the suspicion of cancer, but blood tests cannot be used as a stand-alone tool to rule out cancer.

**Electronic supplementary material:**

The online version of this article (10.1186/s12885-017-3845-9) contains supplementary material, which is available to authorized users.

## Background

Danish cancer patients are diagnosed at a later stage and have a lower cancer survival rate than patients in other western countries [1, 2]. As a result, urgent referral pathways have been introduced for a number of specific cancer types [3, 4]. Referral from primary care to one of these pathways is based on specific alarm symptoms (such as rectal bleeding, dysphagia or breast lump) that are considered suggestive of specific cancer sites [5, 6]. However, only half of patients with cancer present with specific alarm symptoms [7, 8]. The remaining half present with either vague symptoms or non-specific serious symptoms, and they have a longer diagnostic pathway than patients with specific alarm symptoms [7].

An urgent referral pathway for patients with non-specific serious symptoms was developed and implemented at the Diagnostic Centre at Silkeborg Regional Hospital; this pathway was subsequently implemented throughout Denmark in 2011–2012 [9–11]. The diagnostic pathway is intended for patients with non-specific serious symptoms, whom the general practitioner (GP) suspects suffer from a serious disease although the symptoms could be suggestive of a wide range of conditions.

The majority of patients referred to this pathway present with non-specific symptoms, such as weight loss, fatigue and general malaise [12]. Such non-specific symptoms may have several causes and are features of both serious non-malignant disease and cancer. Therefore, the GP first initiates diagnostic workup consisting of imaging and a standardised panel of blood tests as part of the diagnostic pathway. This is referred to as a “triage function” or “filter function”. Within 3 days, the results of the diagnostic tests are delivered to the GP who decides on the next step together with the patient (i.e. watchful waiting, referral to further diagnostic workup or initiation of treatment) [10, 11].

The purpose of the standardised blood test panel is to enable GPs to make a fast diagnostic evaluation of serious disease (serious non-malignant disease and cancer), and the blood tests are selected based on best clinical practice. However, no study has yet addressed the diagnostic value of blood tests among patients referred with non-specific serious symptoms of cancer. As the diagnostic spectrum and prevalence of both non-malignant and malignant disease vary considerably between patients referred with non-specific serious symptoms and the usual patients in primary care, the clinical performance of blood tests may also vary greatly [13]. The aim of the present study was to examine the diagnostic value of the blood test panel used by GPs when cancer is suspected in patients with non-specific serious symptoms.

## Method

### Study design and population

We performed a cohort study that included all patients aged 18 years or older who had been referred by their GP to the blood test panel in the triage function at Silkeborg Regional Hospital in the Central Denmark Region between 1 February 2011 and 31 December 2013. Eligible patients were detected using a specific identifier in the laboratory information system. The unique civil registration number, which is assigned to all Danish citizens at birth or immigration, allowed linkage to the Danish Cancer Registry (DCR) [14]. The DCR contains information about all incident cancers diagnosed from 1943 in the Danish population; these data are coded according to the International Classification of Diseases, 10th revision (ICD-10) [15]. All patients were followed for 3 months in the DCR for a cancer diagnosis (except for non-melanoma skin cancer). Patients were excluded if they had been diagnosed with cancer during the preceding 10 years or had less than 10 valid blood tests.

### Setting

All Danish citizens have free access to the publicly funded healthcare system. Approximately 98% of all citizens in Denmark are registered with a general practice and can thereby consult their GP for medical care when needed [16]. GPs act as gatekeepers to the secondary healthcare system and can initiate diagnostic workup and treatment for most chronic and acute diseases [17].

The urgent pathway for non-specific serious symptoms consists of a two-step approach. Firstly, an initial triage function (imaging and blood test panel) is requested by the GP. Secondly, if relevant, a referral for further diagnostic workup is sent by the GP to the Diagnostic Centre [11]. The imaging may consist of either a computed tomography (CT) scan of the chest, abdomen and pelvis or a combined thoracic X-ray and ultrasound of the upper and lower abdomen. The investigations are performed and the results are reported electronically to the GP within 3 days. On the basis of these investigations, the GP is responsible for taking clinical action and deciding on the ensuing diagnostic approach. If the triage function yields no obvious explanation for a patient’s symptoms, the GP is advised to refer the patient to the Diagnostic Centre. After this referral, the Diagnostic Centre takes over the responsibility for the patient’s diagnostic workup [18].

### Data

A specific identifier was introduced in the laboratory information system at Silkeborg Regional Hospital in January 2011, allowing identification of patients referred by the GP to the blood test panel. We assigned the index date as the date when the blood test panel was requested. Results of all blood tests were registered electronically in the clinical laboratory information system (LABKA) according to the international NPU (Nomenclature, Properties and Units) coding system [19, 20]. For each analysis undertaken, LABKA stored the test result (or indicated that it was missing), the patient’s unique civil registration number, date of blood test analysis and provided the identification number of the referring GP practice. If the GP had requested blood panel tests more than once for the same patient during the inclusion period, only the first tests were included. If results were missing for the first tests, we allowed inclusion of the new tests if the GP had requested the new analysis within 14 days from the first referral using the specific identifier.

### Variables

The blood test panel at Silkeborg Regional Hospital consisted of 48 blood tests (Additional file 1). As some of the tests in the panel were not relevant to cancer diagnostics, we selected 27 laboratory tests for men (22 unspecific tests and 5 tumour markers) and 26 laboratory tests for women (22 unspecific tests and 4 tumour markers) that we hypothesised to be predictive of cancer (Table 1).

**Table 1:** Definition of abnormal blood test results for the 28 included blood tests

Blood test interval		Reference group	Definition of abnormal result
Inflammation			
CRP	High	All	≥8.0 mg/l
ESR	High	Men under 50 years old Men 50 years or older Women under 50 years old Women 50 years or older	≥ 15 mm/h ≥ 20 mm/h ≥ 20 mm/h ≥ 30 mm/h
Immunology			
IgA	Low	All	< 0.8 g/l
	High	Under 50 years old 50 years or older	> 3.90 g/l > 4.90 g/l
IgG	Low	All men, and women 50 years or older Women under 50 years old	< 6.1 g/l < 6.9 g/l
	High	All men, and women 50 years or older Women under 50 years old	> 14.9 g/l l > 15.7 g/l
IgM	Low	All men, and women 50 years or older Women under 50 years old	< 0.39 g/l < 0.55 g/l
	High	All men, and women 50 years or older Women under 50 years old	> 2.08 g/l > 2.30 g/l
Haematology			
White blood cell count	Low	All	< 3.5 × 10^9^/l
	High	All	> 10.0 × 10^9^/l
Neutrophil count	Low	All	< 2.0 × 10^9^/l
	High	All	> 7.0 × 10^9^/l
Eosinophil count	High	All	≥ 0.5 × 10^9^/l
Basophil count	High	All	≥ 0.10 × 10^9^/l
Metamyelocyte count	High	All	≥ 0.05 × 10^9^/l
Monocyte count	Low	All	< 0.2 × 10^9^/l
	High	All	> 0.7 × 10^9^/l
Lymphocyte count	Low	All	< 1.3 × 10^9^/l
	High	All	> 3.5 × 10^9^/l
Platelet count	Low	Men Women	< 145 × 10^9^/l < 165 × 10^9^/l
	High	Men Women	> 350 × 10^9^/l > 400 × 10^9^/l
Anaemia			
Haemoglobin	Low	Men Women	< 8.3 mmol/l < 7.3 mmol/l
Liver and metabolism			
Albumine	Low	Under 70 years old 70 years or older	< 36 g/l < 34 g/l
Alkaline phosphatase	High	All	> 105 U/l
ALK	High	Men Women	> 70 U/l > 45 U/l
Bilirubin	High	All	> 25 μmol/l
Amylase	High	All	> 120 U/l
Calcium total	High	All	> 2.55 mmol/l
LDH	High	Under 70 years old 70 years or older	>205 U/l >255 U/l
Uric acid	High	Men Women under 50 years old Women 50 years or older	>0.48 mmol/l >0.35 mmol/l >0.40 mmol/l
Tumour markers			
M protein	High	All	≥ 2.0 g/l
sFLC ratio	Low	All	< 0.26
	High	All	> 1.65
AFP	High	All	≥ 7 KU/l
PSA	High	Men under 60 years old Men between 60 and 69 years old Men 70 years or older	≥ 3.0 μg/l ≥ 4.0 μg/l ≥ 5.0 μg/l
hCG	High	Men	≥ 2 IU/l
CA-125	High	Women	≥ 35 kIU/l

The blood tests were divided into intervals according to the reference range used at the Department of Clinical Chemistry at Silkeborg Regional Hospital

*Abbreviations: CRP* C-reactive protein, *ESR* erythrocyte sedimentation rate, *IgA* immunoglobulin A, *IgG* immunoglobulin G, *IgM* immunoglobulin M, *ALT* alanine aminotransaminase, *LDH* lactate dehydrogenase. *sFLC* serum-free light chain κ/λ ratio, *AFP* alpha-fetoprotein, *PSA* prostate-specific antigen, *hCG* human chorionic gonadotropin, *CA-125* cancer antigen 125

Abnormal test results were defined as test results that were outside the normal reference range established by the Department of Clinical Chemistry at Silkeborg Regional Hospital (Table 1). We excluded the following blood test results as these intervals were considered irrelevant for cancer diagnosis: high albumin, low amylase, low bilirubin, low alkaline phosphatase, high haemoglobin and low uric acid.

Based on a literature search, we pre-defined seven different abnormal blood tests that we hypothesised would frequently be abnormal in patients diagnosed with cancer [21–29]. These blood tests were used in the analysis of combinations of two abnormal blood tests in cancer diagnostics.

We defined four different types of anaemia based on measurements of haemoglobin, ferritin and C-reactive protein (CRP) [30–32]: 1. Iron deficiency anaemia: anaemia with ferritin <30 microgram/l (μg/l), regardless of CRP level; 2. Anaemia from other causes: anaemia with ferritin >30 μg/l and normal CRP; 3. Combined inflammatory anaemia and iron deficiency anaemia (CIIDA): anaemia with ferritin <100 μg/l and increased CRP; and 4. Inflammatory anaemia: anaemia with ferritin >100 μg/l and increased CRP.

### Statistical analysis

The probability of cancer was calculated as the proportion of patients registered with a new cancer diagnosis in the DCR during the 3 months of follow-up from the index date. A chi-squared test and the Wilcoxon rank sum test were used to test differences between patients diagnosed with and without cancer. For each abnormal blood test, the likelihood ratio (LR) of cancer was calculated. The post-test probability of cancer was calculated in each interval for all abnormal blood tests using Bayes’ theorem [33]. Prior odds were calculated from the prevalence of cancer in the study population (pre-test probability of cancer). For abnormal blood tests, effect measure modification of age (18–64 years old or ≥65 years old) and gender was calculated for the LR of cancer. Only blood tests with an LR of cancer exceeding 1.0 for the total study population and with abnormal results in at least 100 patients were included to ensure reasonable statistical precision. The post-test probability of cancer in patients with different numbers of abnormal blood tests was calculated for five different intervals (0, 1–2, 3–5, 6–8 and ≥9 abnormal blood test results). The 95% confidence intervals (CI) were calculated assuming exact binomial distribution. Data analysis was conducted using Stata Statistical Software version 14.

## Results

### Study population

**Fig. 1:**
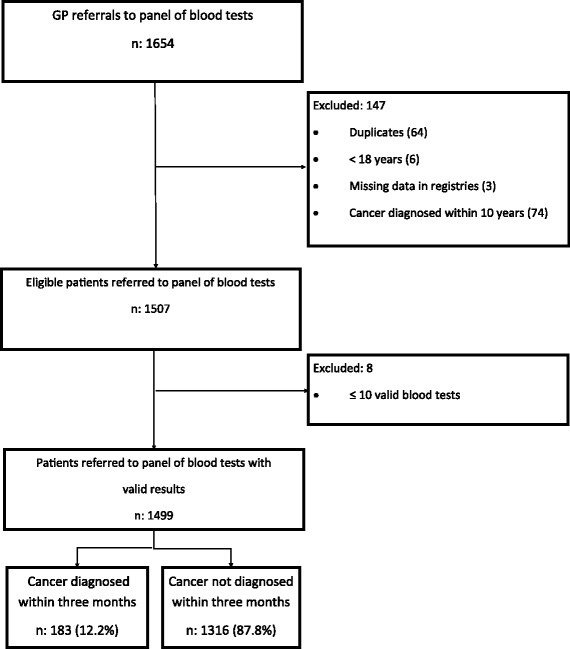
Application of exclusion criteria

A total of 1654 blood test panels were requested by GPs, and 1499 (90.6%) patients complied with the inclusion criteria (Fig. 1). After 3 months of follow-up, 183 patients (12.2%) were diagnosed with cancer (Table 2). These patients were more likely to be older and male than patients not diagnosed with cancer. The most frequently diagnosed malignancies were lung cancer, colorectal cancer, haematological cancers, prostatic cancer and pancreatic cancer (Additional file 2). The tumour stage (local vs. regional/distant) was local in 29/88 of patients (33%) diagnosed with solid cancer (the tumour stage was missing in the DCR for 64 patients diagnosed with solid cancer).

**Table 2:** Baseline characteristics of patients referred by GPs to the panel of blood tests as part of the triage function (*n* = 1499)

	Patients diagnosed with cancer (n = 183, 12.2%)	Patients not diagnosed with cancer (*n* = 1316, 87.8%)	*p*-value
Age, years			
median (IQI)	71 (66–77)	65 (54–75)	<0.001^a^
Gender, n (%)			
Male	108 (59.0)	606 (46.0)	<0.001^b^
Female	75 (41.0)	710 (54.0)	
Number of valid blood test results (median (IQI))			
Men	27 (25–27)	26 (25–27)	0.110^a^
Women	26 (24–26)	25 (24–26)	0.392^a^

^a^Wilcoxon rank sum test

^b^Chi-squared test

### Abnormal blood test results and post-test probability of cancer

**Fig. 2:**
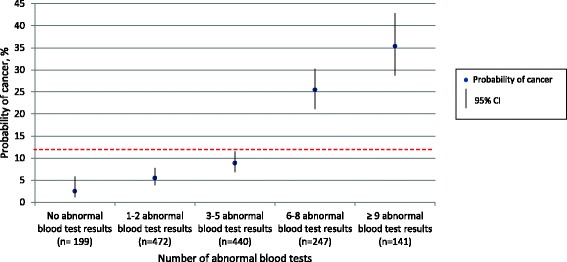
Number of abnormal blood tests and probability of cancer. The red line indicates the pre-test probability of cancer

The median number of abnormal blood tests was 7 (interquartile interval (IQI): 4–10) for patients diagnosed with cancer and 3 (IQI: 1–6) for patients not diagnosed with cancer (*p* < 0.001) (data not shown). There was a markedly increased probability of cancer with six or more abnormal blood tests (probability of cancer_6–8 abnormal blood tests_ = 25.5% and probability of cancer ≥_9 abnormal blood tests_ = 35.4%); less than two abnormal blood tests lowered the post-test probability of cancer (Fig. 2).

The most frequent abnormal blood tests among cancer patients were high inflammatory markers (CRP or erythrocyte sedimentation rate (ESR)), high monocyte count, anaemia, low lymphocyte count, hypo-albuminaemia and high alkaline phosphatase (Table 3).

**Table 3:** Sensitivity, specificity, likelihood ratio and post-test probability of cancer for the 28 different blood tests

Blood test interval	Valid blood tests (n)	Number of abnormal test results (n (%))	Sensitivity/specificity	Likelihood ratio of cancer (95% CI)	Post-test probability of cancer (95% CI)^†^
Inflammation					
High CRP	1474	438 (30%)	0.60/0.74	2.4 (2.0; 2.7)	24.7 (21.9; 27.6)
High ESR	1154	375 (32%)	0.61/0.72	2.2 (1.9; 2.6)	23.3 (20.6; 26.3)
Immunology					
IgA	1473				
Low		46 (3%)	0.08/0.98	3.1 (1.7; 5.7)	30.1 (19.0; 44.2)
High		108 (7%)	0.10/0.93	1.4 (0.9; 2.3)	16.5 (10.9; 24.2)
IgG	1472				
Low		78 (5%)	0.08/0.95	1.7 (1.0; 2.9)	19.1 (12.1; 28.8)
High		71 (5%)	0.08/0.96	1.8 (1.0; 3.1)	19.6 (12.2; 30.0)
IgM	1474				
Low		114 (8%)	0.13/0.91	1.4 (0.9; 2.1)	16.5 (11.6; 22.9)
High		123 (8%)	0.12/0.92	1.6 (1.0; 2.4)	17.7 (12.2; 24.9)
Haematology					
White blood cell count	1486				
Low		20 (1%)	0.01/0.99	0.4 (0.1; 2.8)	5.0 (0.7; 28.1)
High		226 (15%)	0.24/0.86	1.7 (1.3; 2.3)	19.5 (15.3; 24.5)
Neutrophil count	1469				
Low		56 (4%)	0.02/0.96	0.6 (0.2; 1.6)	7.4 (2.8; 17.8)
High		204 (14%)	0.29/0.88	2.5 (1.8; 3.3)	25.6 (20.7; 31.2)
High eosinophil count	1469	62 (4%)	0.06/0.96	1.6 (0.9; 3.0)	18.2 (10.6; 29.6)
High basophil count	1468	41 (3%)	0.03/0.97	1.0 (0.4; 2.6)	12.6 (5.4; 26.5)
High metamyelocytes	1468	161 (11%)	0.24/0.91	2.5 (1.9; 3.5)	26.1 (20.5; 32.7)
Monocyte count	1468				
Low		13 (1%)	0.06/0.99	0.6 (0.1; 4.7)	7.9 (1.1; 39.7)
High		473 (32%)	0.51/0.70	1.7 (1.4; 2.0)	19.1 (16.6; 21.9)
Lymphocyte count	1469				
Low		360 (25%)	0.42/0.78	1.9 (1.6; 2.3)	20.8 (17.7; 24.4)
High		42 (3%)	0.02/0.97	0.8 (0.3; 2.2)	9.8 (3.8; 23.2)
Platelet count	1481				
Low		72 (5%)	0.10/0.96	2.4 (1.5; 4.0)	25.1 (16.7; 35.8)
High		210 (14%)	0.25/0.87	2.0 (1.5; 2.6)	21.5 (17.0; 26.8)
Anaemia					
Low haemoglobin	1482	428 (29%)	0.48/0.74	1.8 (1.5; 2.2)	20.1 (17.4; 23.1)
Iron deficiency anaemia	1470	83 (6%)	0.08/0.95	1.4 (0.8; 2.5)	16.7 (10.4; 25.9)
Anaemia, other causes	1452	145 (10%)	0.11/0.89	1.1 (0.7; 1.7)	12.9 (8.7; 18.7)
Combined anaemia	1452	25 (2%)	0.04/0.98	2.8 (1.2; 6.7)	28.3 (14.3; 48.2)
Inflammatory anaemia	1452	159 (11%)	0.25/0.91	2.8 (2.1; 3.8)	28.0 (22.2; 34.6)
Liver and metabolism					
Low albumin	1494	361 (24%)	0.41/0.78	1.9 (1.5; 2.3)	20.7 (17.6; 24.2)
High ALT	1484	118 (8%)	0.12/0.93	1.7 (1.1; 2.6)	18.7 (13.0; 26.3)
High alkaline phosphatase	1479	257 (17%)	0.37/0.85	2.5 (2.0; 3.2)	26.0 (21.8; 30.7)
High bilirubin	1479	38 (3%)	0.07/0.98	3.3 (1.7; 6.4)	31.5 (19.1; 47.2)
High amylase^c^	1070	51 (5%)	0.04/0.95	0.9 (0.4; 2.0)	10.7 (5.0; 21.7)
High calcium	1484	119 (8%)	0.18/0.93	2.8 (1.9; 4.0)	27.7 (21.0; 35.7)
High LDH	1429	160 (11%)	0.22/0.90	2.3 (1.6; 3.1)	23.9 (18.5; 30.4)
High uric acid	1484	119 (8%)	0.09/0.92	1.1 (0.7; 1.9)	13.4 (8.6; 20.4)
Tumour markers					
High M-protein^c^	1050	33 (3%)	0.09/0.98	4.3 (2.2; 8.4)	37.4 (23.3; 54.0)
sFLC ratio	1455				
Low		30 (2%)	0.05/0.98	2.6 (1.2; 5.8)	26.7 (12.3; 45.9)
High		201 (14%)	0.16/0.87	1.2 (0.8; 1.7)	13.9 (9.5; 19.5)
High AFP	1446	159 (11%)	0.10/0.89	0.9 (0.6; 1.4)	11.0 (7.2; 16.4)
High PSA^a^	694	97 (14%)	0.24/0.88	1.9 (1.3; 2.9)	21.1 (15.1; 28.6)
High hCG^a^	684	37 (5%)	0.18/0.97	5.8(3.1; 10.6)	44.4 (30.3; 59.6)
High CA-125^b^	769	93 (12%)	0.39/0.91	4.2 (2.9; 6.1)	36.8 (28.7; 45.7)

*Abbreviations*: *ESR* erythrocyte sedimentation rate, *CRP* C-reactive protein, *IgA* immunoglobulin A, *IgG* immunoglobulin G, *IgM* immunoglobulin M, *ALT* alanine aminotransaminase, *LDH* lactate dehydrogenase, *sFLC* serum-free light chain κ/λ ratio, *AFP* alpha-fetoprotein, *PSA* prostate-specific antigen, *hCG* human chorionic gonadotropin, *CA-125* cancer antigen 125

^†^The pretest probability of cancer was 12.2%; a likelihood ratio of >1.0 increased the probability of cancer. ^a^PSA and hCG were only performed in men

^b^CA-125 was only performed in women

^c^As the reference range was changed for M protein and amylase during the inclusion period by the Department of Clinical Chemistry at Silkeborg Regional Hospital, we did not include test results for these blood tests after this change

Twenty-five of the blood tests had an estimated LR of cancer above 1.0 when abnormal (Table 3), which resulted in post-test probabilities of cancer ranging from 13.4 to 44.4%. The highest post-test probability of cancer was found in patients with high human chorionic gonadotropin (hCG) (44.4%), high M protein (37.4%) or high cancer antigen 125 (CA-125) (36.8%). In men with high hCG and women with high CA-125, the predominant cancer types were of non-gonadal origin.

Besides abnormal tumour markers, cancer was seen in more than 25% of patients with high bilirubin, low immunoglobulin A (IgA), high calcium, high metamyelocyte count, high alkaline phosphatase, high neutrophil count or low platelet count (Table 3). Among cancer patients with low IgA, 54% were diagnosed with malignant plasma cell disorders. For anaemia, the probability of cancer varied between the different anaemia types; the highest post-test probability of cancer was found among patients with either CIIDA (28%) or inflammatory anaemia (28%).

For high calcium and inflammatory anaemia, the LR of cancer was most markedly increased in patients aged less than 65 years (high calcium: LR of cancer_age group 18–64 years_ = 7.3 vs. LR of cancer_age group ≥ 65 years_ = 1.7 and inflammatory anaemia: LR of cancer_age group 18–64 years_ = 4.4 vs. LR of cancer_age group ≥ 65 years_ = 1.9) (Additional file 3). Gender had no significant influence on the LR of cancer (Additional file 3).

Table 4 shows the post-test probability of cancer for combinations of two abnormal blood tests. All combinations resulted in a more than twofold increased probability of cancer, especially combinations of two abnormal blood tests including high calcium.

**Table 4:** Post-test probability of cancer for combinations of two abnormal blood tests

	High LDH	High alkaline phosphatase	Thrombocytosis	High WBC count	High CRP	Anaemia
Hypercalcaemia	62.8 (38.3; 82.1) (*n* = 16)	46.6 (32.2; 61.7) (*n* = 39)	46.8 (30.4; 63.9) (*n* = 30)	47.1 (31.7; 63.2) (*n* = 34)	42.6 (31.5; 54.5) (*n* = 62)	34.1 (22.0; 48.6) (*n* = 44)
Anaemia	26.2 (17.5; 37.2) (*n* = 69)	29.9 (22.6; 38.3) (*n* = 111)	23.9 (17.7; 31.4) (*n* = 126)	28.5 (20.0; 39.0) (*n* = 77)	28.9 (23.8; 34.6) (*n* = 200)	
High CRP	36.6 (26.4; 48.1) (*n* = 67)	34.5 (28.0; 41.7) (*n* = 145)	30.3 (23.8; 37.6) (*n* = 138)	26.6 (20.7; 33.5) (n = 149)		
High WBC count	34.6 (20.6; 52.8) (*n* = 29)	27.8 (19.3; 38.3) (*n* = 76)	28.7 (20.4; 38.6) (*n* = 84)			
Thrombocytosis	37.8 (21.2; 57.7) (*n* = 24)	26.4 (17.4; 37.9) (*n* = 65)				
High alkaline phosphatase	30.9 (21.3; 42.4) (*n* = 66)					

The post-test probability of cancer is shown in each cell with the 95% CI shown in parenthesis. The number of patients with each combination is shown at the bottom of each cell. Pre-test probability of cancer: 12.2%

*Abbreviations*: *LDH* lactate dehydrogenase, *CRP: C*-reactive protein, *WBC count* white blood cell count

## Discussion

### Main findings

This study is the first to quantify the diagnostic value of routine blood tests in patients referred from general practice through an urgent referral route for patients with non-specific serious symptoms. Twelve percent of patients referred by the GP were diagnosed with cancer. The probability of cancer increased with a growing number of abnormal blood tests, and combinations of two abnormal blood tests indicated a 23–62% probability of cancer. For single blood tests, a number of specific abnormal test results yielded a high probability of cancer, but most single-test results yielded only a small increase in the post-test probability of cancer.

### Comparison with previous studies and discussion of findings

In our study population, the risk of cancer was 12%. Our figure was thus lower than the 16% reported in a previous study [12]. However, this previous study did not exclude patients who had suffered from cancer within the past 10 years. The study found that the symptoms with the highest LR of cancer among patients referred by their GP was jaundice (LR: 3.9), dysphagia (LR: 3.0) and lump (LR: 1.9). Still, with the exception of patients presenting with a lump, most of these symptoms were infrequent among patients referred by their GP to the triage function (present in less than 2% of patients). This study also demonstrated that approximately 60% of patients examined in the triage function were later referred to the diagnostic centre by their GP.

A recent Danish study of patients referred by their GP for diagnostic workup at a diagnostic centre found proportions of anaemia, high ESR and alkaline phosphatase in patients who were later diagnosed with cancer that were similar to those reported in our study [34]. This study used the same cut-points as our study, and the authors found a LR of cancer of 1.8 among patients with anaemia, a result that is very similar to our findings.

The prevalence of inflammatory anaemia in cancer patients has been reported to range between 30 and 77% [35]. In line with this finding, we demonstrated a high probability of cancer among patients with inflammatory anaemia. We are unaware of other publications that explore the value of inflammatory anaemia in cancer diagnostics.

The association between hypercalcaemia and malignant disease is well established in both primary and secondary care studies. A UK case-control study in primary care found that even mild hypercalcaemia had a PPV for cancer of 11.5% in men and 4.1% in women [29]. In our study, 27% of patients with hypercalcaemia were diagnosed with cancer; the risk was even higher in hypercalcaemic patients with high levels of one of the following: lactate dehydrogenase (LDH), alkaline phosphatase, white blood cell count, CRP or platelet count. Even though the prevalence of cancer is much lower in primary care, the results highlight the importance of hypercalcaemia in cancer diagnostics. Still, as hypercalcaemia has been associated with poor prognosis in cancer patients, its occurrence may be a sign of advanced disease [36]. The diagnostic value of hypercalcaemia in early cancer diagnostics may accordingly be limited.

### Strengths and limitations

Key strengths of this study were the large number of patients who were included and the prospective data collection combined with a standardised panel of blood tests that remained the same throughout the study period. Furthermore, quantification of the diagnostic value of blood tests in a patient population suspected of having a wide variety of diseases increases the usefulness of the results in clinical practice.

The specific identifier was unique for the panel of blood tests. Consequently, the specificity of this method for identifying referred patients was high. However, it was not possible to use the specific identifier if GPs had not installed the latest version of the software for handling blood tests when ordering online. Thus, we may have missed some patients, especially at the beginning of the inclusion period. Additionally, GPs were allowed to deselect actively blood samples (including the specific identifier). We had no method to estimate the number of patients who were missed due to deselection of blood samples, but we consider such deselections to be rare.

The register data are generally considered valid and complete as information in the DCR is continuously updated based on national registries, and the records have been shown to be highly accurate [15]. The same applies to the LABKA system, which includes all clinical information on blood test results in the Central Denmark Region. We used a follow-up period of 3 months as we wanted to include the incident cancers that had caused the symptoms that made the GP request the blood test panel. This follow-up interval was based on a marked decline in the number of patients registered with a new cancer 3 months after the index date (12 patients were diagnosed with a new cancer 4–6 months after the index date, and 15 patients were diagnosed with a new cancer 7–12 months after the index date). However, we had no information on reasons why the patients encountered their GP, and some cancer types, such as prostate cancer, may have accidentally been diagnosed using a standardised blood test panel. Still, non-specific symptoms may be features of several cancer types, including prostate cancer [37, 38]. Furthermore, GPs interpreted the results of the blood test panel based on the patients’ medical history and clinical findings, and decided on further diagnostic tests accordingly. However, we are not able to reject the possibility that using multiple blood tests may have caused accidental diagnosis of some of the cancers in the study.

An important misclassification of blood test results may be due to other known diseases. We did not include information about known comorbidity or severity of disease in patients that may have caused some of the blood tests to be abnormal. Furthermore, we studied the diagnostic value of blood tests when the test results were abnormal compared to the reference range of the Department of Clinical Biochemistry. Thus, the reference values of our test panel were not specific for cancer. More research is needed to evaluate the importance of earlier blood test results and identify appropriate cut-off values for cancer diagnosis.

We used a simple diagnostic algorithm to define subtypes of anaemia [31] although more complex algorithms for establishing the cause of anaemia have been reported [32]. Still, a ferritin level below 30 μg/l has high diagnostic accuracy for iron-deficiency anaemia [30, 31, 39]. However, under inflammatory conditions, the serum ferritin level may be a poor marker of iron stores, and other laboratory markers may thus be required (e.g. soluble transferrin receptor or ferritin index). Still, as no universally accepted diagnostic algorithm allows differentiation between CIIDA and inflammatory anaemia [32], we used an algorithm that is clinically applicable for GPs.

### Implications for clinical practice

The purpose of the blood test panel is to enable GPs to make fast clinical decisions on further diagnostic workup for patients with non-specific serious symptoms of disease. We found that the information derived from a number of specific abnormal blood tests markedly increased the probability of finding cancer, although none of the abnormal blood tests were specific to cancer.

Our findings suggest that further diagnostic investigations for cancer are warranted especially in patients with multiple abnormal blood test results and in patients with certain combinations of abnormal test results. While specific abnormal blood tests may be highly predictive of cancer (e.g. high hCG, M protein or high bilirubin), single abnormal test results seem to be of little value in cancer diagnostics. Still, the risk of cancer is high among patients referred to the triage function. Therefore, given the non-specificity of the blood tests, GPs should not hesitate to refer patients for further investigation if no obvious explanation is present for patients’ symptoms after the triage function.

Apart from cancer, GPs may consider other nonmalignant conditions, especially in combination with the imaging results that form part of the triage function. Our results emphasise the importance of strong interaction and integration between primary and secondary care regarding diagnostic pathways for these patients.

## Conclusion

Relatively few abnormal blood tests increased the risk of cancer in patients referred to the triage function. Specific combinations of two abnormal blood tests increased the risk of cancer, but most of these combinations were rarely present. The blood test panel should be interpreted with caution if cancer is suspected. Further diagnostic workup is warranted if no obvious explanation for a patient’s symptoms is found after the triage function.

## Additional files

Additional file 1: An overview of the blood tests used in the triage function at Silkeborg Regional Hospital. Forty-eight different blood tests were used in the triage function. The highlighted blood tests represent the 28 blood tests included in the present study. Abbreviations: CRP: C-reactive protein; ESR: erythrocyte sedimentation rate; IgA: immunoglobulin A; IgG: immunoglobulin G; IgM: immunoglobulin M; MCHC: mean corpuscular haemoglobin concentration; ALT: alanine amino-transaminase; INR: international normalised ratio; LDH: lactate dehydrogenase; TSH: thyroid-stimulating hormone; HBA1C: glycated haemoglobin; sFLC: serum-free light chain κ/λ ratio; AFP: alpha-fetoprotein; hCG: human chorionic gonadotropin; PSA: prostate-specific antigen; CA-125: cancer antigen 125; ANA: antinuclear antibody. (XLSX 10 kb)

Additional file 2: Cancer types (*n* = 183 patients). *: Haematological cancers included 12 patients with lymphoma, 12 patients with malignant plasma cell disorders and 8 patients with leukemia.**: “Other cancer types” refers to cancer types diagnosed in less than five patients and included the following cancer types: bladder cancer, breast cancer, central nervous system cancer, female reproductive cancer, head and neck cancer, soft tissue cancer and malignant melanoma. (PDF 150 kb)

Additional file 3: Effect measure modification of gender and age on likelihood ratio of cancer. Pre-test probability of cancer: All patients = 12.2%; age-group 18–64 years = 5.9%, age-group ≥65 years = 18.0%, males = 15.1%, and females = 9.6%. Abbreviations: CRP: C-reactive protein, ESR: erythrocyte sedimentation rate; ALK: alanine aminotransaminase. (XLSX 14 kb)

## Abbreviations

CA-125: Cancer antigen 125
CI: Confidence interval
CIIDA: Combined inflammatory anaemia and iron deficiency anaemia
CRP: C-reactive protein
CT: Computed tomography
DCR: Danish Cancer Registry
ESR: Erythrocyte sedimentation rate
GP: General practitioner
hCG: human chorionic gonadotropin
ICD-10: International Classification of Diseases, 10th revision (ICD-10)
IgA: Immunoglobulin A
IQI: Interquartile interval
LABKA: Clinical laboratory information system
LDH: Lactate dehydrogenase
LR: Likelihood ratio
NPU: Nomenclature, Properties and Units
μg/l: microgram/l

## References

[1] Coleman MP, Forman D, Bryant H, Butler J, Rachet B, Maringe C (2011). Cancer survival in Australia, Canada, Denmark, Norway, Sweden, and the UK, 1995-2007 (the international cancer benchmarking partnership): an analysis of population-based cancer registry data. Lancet.

[2] De Angelis R, Sant M, Coleman MP, Francisci S, Baili P, Pierannunzio D (2014). Cancer survival in Europe 1999-2007 by country and age: results of EUROCARE--5-a population-based study. Lancet Oncol.

[3] National Board of Health: National Cancer Plan II (2005). Denmark National Board of health recommendation for improving cancer health services.

[4] Probst HB, Hussain ZB, Andersen O (2012). Cancer patient pathways in Denmark as a joint effort between bureaucrats, health professionals and politicians--a national Danish project. Health Policy.

[5] Shapley M, Mansell G, Jordan JL, Jordan KP (2010). Positive predictive values of >/=5% in primary care for cancer: systematic review. Br J Gen Pract.

[6] Neal RD, Din NU, Hamilton W, Ukoumunne OC, Carter B, Stapley S (2014). Comparison of cancer diagnostic intervals before and after implementation of NICE guidelines: analysis of data from the UK general practice research database. Br J Cancer.

[7] Jensen H, Torring ML, Olesen F, Overgaard J, Vedsted P (2014). Cancer suspicion in general practice, urgent referral and time to diagnosis: a population-based GP survey and registry study. BMC Cancer.

[8] Nielsen TN, Hansen RP, Vedsted P (2010). Symptom presentation in cancer patients in general practice. Ugeskr Laeger.

[9] The National Board of Health (2012). Diagnostic pathway for patients with non-specific symptoms of serious illness that might be cancer.

[10] Vedsted P, Olesen F (2015). A differentiated approach to referrals from general practice to support early cancer diagnosis - the Danish three-legged strategy. Br J Cancer.

[11] Fredberg U, Vedsted P (2011). Organisation of diagnosing patients with unspecific cancer symptoms. Ugeskr Laeger.

[12] Ingeman ML, Christensen MB, Bro F, Knudsen ST, Vedsted P (2015). The Danish cancer pathway for patients with serious non-specific symptoms and signs of cancer-a cross-sectional study of patient characteristics and cancer probability. BMC Cancer.

[13] Usher-Smith JA, Sharp SJ, Griffin SJ (2016). The spectrum effect in tests for risk prediction, screening, and diagnosis. BMJ.

[14] Schmidt M, Pedersen L, Sorensen HT (2014). The Danish civil registration system as a tool in epidemiology. Eur J Epidemiol.

[15] Gjerstorff ML (2011). The Danish cancer registry. Scand J Public Health.

[16] Andersen JS, Olivarius Nde F, Krasnik A (2011). The Danish National Health Service Register. Scand J Public Health.

[17] Pedersen KM, Andersen JS, Sondergaard J (2012). General practice and primary health care in Denmark. J Am Board Fam Med.

[18] Naeser E, Fredberg U, Moller H, Vedsted P. Clinical characteristics and risk of serious disease in patients referred to a diagnostic centre: a cohort study. Cancer Epidemiol. 2017; 10.1016/j.canep.2017.07.014. [Epub ahead of print] 10.1016/j.canep.2017.07.014 28781173

[19] Grann AF, Erichsen R, Nielsen AG, Froslev T, Thomsen RW (2011). Existing data sources for clinical epidemiology: the clinical laboratory information system (LABKA) research database at Aarhus University, Denmark. Clin Epidemiol.

[20] Pontet F, Magdal Petersen U, Fuentes-Arderiu X, Nordin G, Bruunshuus I, Ihalainen J (2009). Clinical laboratory sciences data transmission: the NPU coding system. Stud Health Technol Inform.

[21] Ludwig H, Van Belle S, Barrett-Lee P, Birgegard G, Bokemeyer C, Gascon P (2004). The European cancer Anaemia survey (ECAS): a large, multinational, prospective survey defining the prevalence, incidence, and treatment of anaemia in cancer patients. Eur J Cancer.

[22] Steinberg D (1989). Anemia and cancer. CA Cancer J Clin.

[23] Levin J, Conley CL (1964). Thrombocytosis associated with malignant disease. Arch Intern Med.

[24] Bailey SE, Ukoumunne OC, Shephard E, Hamilton W (2016). How useful is thrombocytosis in predicting an underlying cancer in primary care? A systematic review. Fam Pract.

[25] Shoenfeld Y, Tal A, Berliner S, Pinkhas J: Leukocytosis in non hematological malignancies--a possible tumor-associated marker. J Cancer Res Clin Oncol 1986, 111(1):54-58. 10.1007/BF00402777 PMC12253059 3949851

[26] Huijgen HJ, Sanders GT, Koster RW, Vreeken J, Bossuyt PM (1997). The clinical value of lactate dehydrogenase in serum: a quantitative review. Eur J Clin Chem Clin Biochem.

[27] Schwartz MK (1973). Enzymes in cancer. Clin Chem.

[28] Heikkila K, Ebrahim S, Lawlor DA (2007). A systematic review of the association between circulating concentrations of C reactive protein and cancer. J Epidemiol Community Health.

[29] Hamilton F, Carroll R, Hamilton W, Salisbury C (2014). The risk of cancer in primary care patients with hypercalcaemia: a cohort study using electronic records. Br J Cancer.

[30] Guyatt GH, Oxman AD, Ali M, Willan A, McIlroy W, Patterson C (1992). Laboratory diagnosis of iron-deficiency anemia: an overview. J Gen Intern Med.

[31] Dahlerup JF, Eivindson M, Jacobsen BA, Jensen NM, Jorgensen SP, Laursen SB (2015). Diagnosis and treatment of unexplained anemia with iron deficiency without overt bleeding. Dan Med J.

[32] Nairz M, Theurl I, Wolf D, Weiss G (2016). Iron deficiency or anemia of inflammation? : Differential diagnosis and mechanisms of anemia of inflammation. Wien Med Wochenschr.

[33] Sox H, Higgins MC, Douglas KW (2013). Medical Decision Making.

[34] Bislev LS, Bruun BJ, Gregersen S, Knudsen ST (2015). Prevalence of cancer in Danish patients referred to a fast-track diagnostic pathway is substantial. Dan Med J.

[35] Weiss G, Goodnough LT (2005). Anemia of chronic disease. N Engl J Med.

[36] Ralston SH, Gallacher SJ, Patel U, Campbell J, Boyle IT (1990). Cancer-associated hypercalcemia: morbidity and mortality. Clinical experience in 126 treated patients. Ann Intern Med.

[37] Hamilton W, Sharp DJ, Peters TJ, Round AP (2006). Clinical features of prostate cancer before diagnosis: a population-based, case-control study. Br J Gen Pract.

[38] Hamilton W (2009). The CAPER studies: five case-control studies aimed at identifying and quantifying the risk of cancer in symptomatic primary care patients. Br J Cancer.

[39] Mast AE, Blinder MA, Gronowski AM, Chumley C, Scott MG (1998). Clinical utility of the soluble transferrin receptor and comparison with serum ferritin in several populations. Clin Chem.

