# Supplementary figures and images for: Routine blood tests and probability of cancer in patients referred with non-specific serious symptoms: a cohort study
Source: BMC Cancer. 2017 Dec 4;17:817. doi: 10.1186/s12885-017-3845-9 (PMC5715646; doi:10.1186/s12885-017-3845-9)

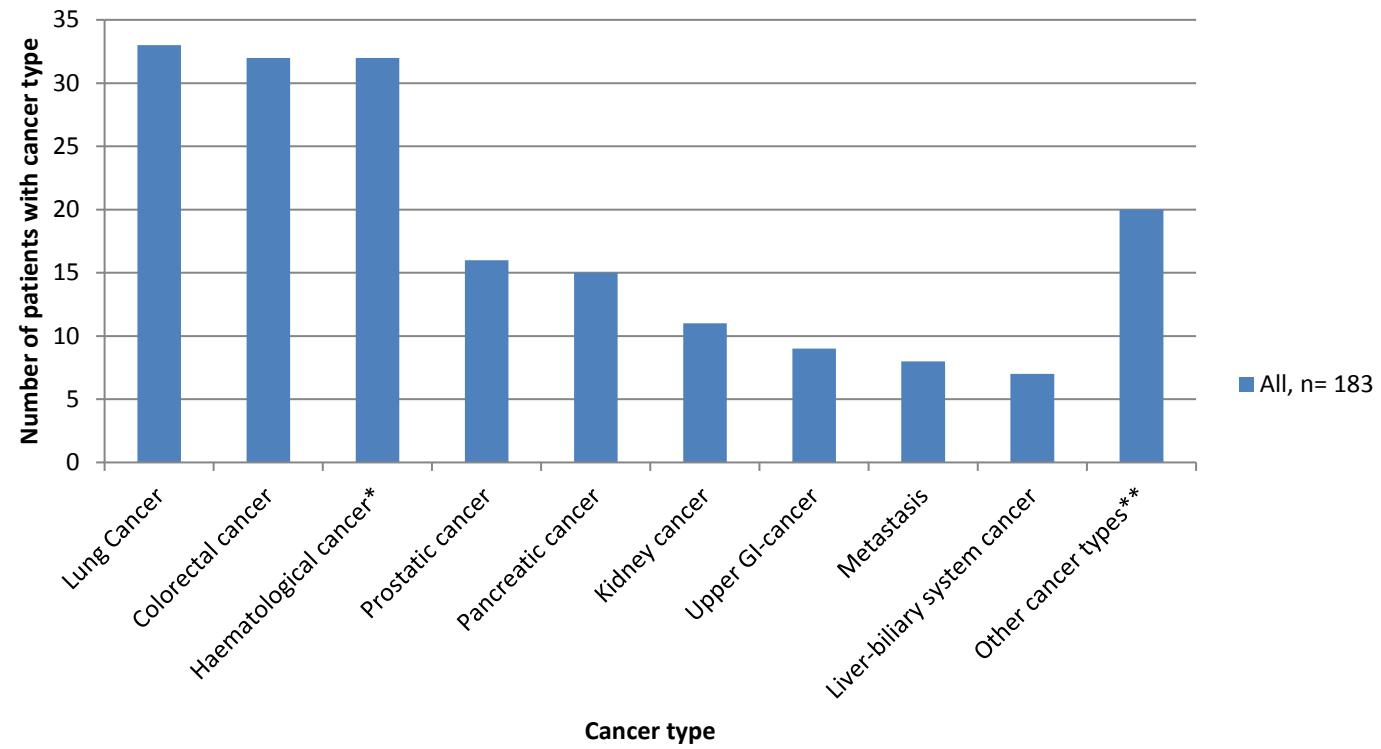

Supplement: Supplementary file 2 — Cancer types (n = 183 patients). *: Haematological cancers included 12 patients with lymphoma, 12 patients with malignant plasma cell disorders and 8 patients with leukemia.**: “Other cancer types” refers to cancer types diagnosed in less than five patients and included the following cancer types: bladder cancer, breast cancer, central nervous system cancer, female reproductive cancer, head and neck cancer, soft tissue cancer and malignant melanoma. (PDF 150 kb) [file 12885_2017_3845_MOESM2_ESM.pdf]
